# Investigating the clinical significance of EGFR expression using machine learning in a series of Iraqi patients with triple-negative breast cancer

**DOI:** 10.25122/jml-2021-0401

**Published:** 2022-08

**Authors:** Gufran Salman, Esraa Aldujaily, Mohammed Jabardi, Omar Layth Qassid

**Affiliations:** 1 Department of Basic Science, Faculty of Dentistry, University of Kufa, Kufa, Iraq; 2 Department of Pathology and Forensic Medicine, Faculty of Medicine, University of Kufa, Kufa, Iraq; 3 Department of Computer Science, College of Education, University of Kufa, Kufa, Iraq; 4 Cancer Research Center, University of Leicester, Leicester City, United Kingdom

**Keywords:** TNBC, EGFR, machine learning, TNBC – Triple-negative breast cancer, EGFR – epidermal growth factor receptor, PCA – principal component analysis, ROC – receiver operator characteristic

## Abstract

Breast cancer is a heterogeneous disease with a distinct profile of the expression of each tumor. Triple-negative breast cancer (TNBC) is a molecular subtype of breast cancer characterized by an aggressive clinical behavior linked to loss or reduced expression of estrogen, progesterone, and Her2/neu receptors. The study's main objective was to investigate the clinical significance of epidermal growth factor receptor (EGFR) overexpression in a series of Iraqi patients with TNBC. The sectional analytic study involved immunohistochemical analysis of EGFR expression in randomly selected 53 formalin fixed paraffin embedded tissue blocks of TNBC cases out of 127 Iraqi patients with TNBC and correlated expression data with clinicopathological parameters including survival time. Machine learning (statistical tests and principal component analysis (PCA)) was used to predict the outcome of the patients using EGFR expression data together with clinicopathological parameters. EGFR was expressed in approximately 28% of TNBC cases. We estimated the risk of mortality and distant metastasis based on EGFR expression and clinicopathologic factors using the principal component analysis (PCA) model. We found a substantial positive correlation between clinical stage and distant metastasis, clinical stage and death, death and distant metastasis, and death and positive EGFR expression. Overall, EGFR expression was linked to a poor prognosis and increased mortality. A higher risk of distant metastasis and death was associated with an advanced clinical stage of the tumor. Furthermore, the existence of distant metastases increased the risk of death. These findings raise the possibility of using EGFR expression data with other clinicopathological parameters to predict the outcome of patients with TNBC.

## INTRODUCTION

The morbidity of breast cancer is still high, and according to the most recent estimates of GLOBCAN 2020, the annual mortality rate is 684 996 worldwide [1]. In the Middle East, the death rate per year is 684,996, and the highest death rates are in low-income countries [1, 2]. Iraq is among the middle east countries with the highest mortality rate of breast cancer, about 20.4/100000 [3, 4]. Triple-negative breast cancer (TNBC) makes up 20% of all breast cancers. TNBC has a more threatening clinical course than non-TNBC. TNBC is recognized by the nonexistence (or minimum expression) of estrogen and progesterone receptors (ER and PR), as well as the inadequacy of HER2 overexpression, according to immunohistochemistry (IHC) [4]. The treatment of TNBC is challenging because of its aggressive behavior and clinical course. Many types of research were carried out to uncover new prognostic and therapeutic targets in TNBC. A four-component algorithm used to generate a single predictor using ER, PR, HER2, and Ki67 was shown to have a predictive value similar to that of Oncotype diagnosis, a commercial microarray assay to estimate the probability of disease recurrence in ER-positive tumors [5].

Epidermal growth factor receptor (EGFR) is a prototypic receptor tyrosine kinase with key roles in epithelial cells. It is a transmembrane protein comprising an extracellular ligand-binding domain, transmembrane domain, and a cytoplasmic tyrosine kinase domain [6, 7]. Activating many downstream signaling pathways, such as Ras-Raf-MEK-ERK, PI3K-AKT-mTOR, and Src-STAT3, enhances cell proliferation, motility, and survival [8]. Studies show that EGFR function can be disrupted by oncogenic mutations or copy number amplification, resulting in EGFR overexpression. EGFR overexpression, which occurs as a result, plays an essential role in tumor generation and progression, especially in highly malignant carcinomas [9]. EGFR is a popular pharmacological target, and EGFR inhibitors, including TKIs and mAbs, have been developed, with some being utilized in clinical trials [10]. Patients with colon carcinoma, non-small cell lung cancer (NSCLC), and squamous-cell head and neck carcinoma who received anti-EGFR therapy lived longer [11]. When it comes to breast cancer, TNBC has a higher rate of EGFR overexpression than other subcategories [12]. In TNBC, EGFR expression, gene amplification, and mutation status have been broadly studied.

The frequency of EGFR protein expression in TNBC interrogation by the IHC showed that a range of 13–76% depends mainly on evaluation methods and antibody methods [13]. Gene expression profiling discovered an epidermal growth factor receptor (EGFR) gene as a biomarker for at least half of individuals with TNBC, laying the groundwork for clinical trials of treatments that target this receptor [14]. However, EGFR is still not approved by the Food and Drug Administration (FDA) as a targeted therapy for TNBC, necessitating intensive research intervention to explore more about the clinical significance of this biomarker.

The main objective of this study was to investigate the clinical significance of EGFR expression in relation to clinicopathological parameters using machine learning in a series of Iraqi patients with TNBC.

## Material and methods

### Study design and setting

**Figure 1:**
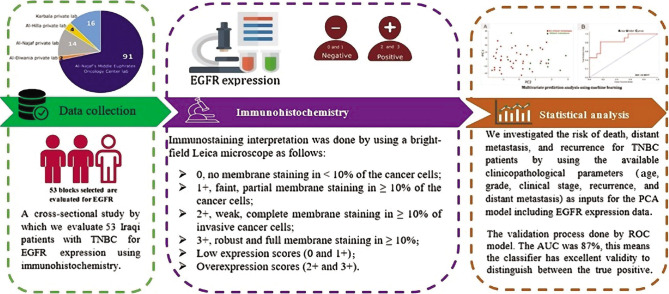
Block diagram of the proposed study.

The study utilized a cross-sectional analytic study design to determine the profile of EGFR immunohistochemical expression and its clinical significance in a series of Iraqi patients with TNBC. The block diagram of this study was divided into three tasks: data collection and preprocessing, immunohistochemistry and interpretation, and statistical analysis (Figure 1).

### Patient Selection

In our study, most TNBC cases were selected from patients treated in a tertiary cancer care center in Iraq's Middle Euphrates Area (MEA). The cancer care center is a referral center for towns along the Middle Euphrates and accepts in-patients and outpatients from around five jurisdictions. We also included patients from private laboratories in MEA towns. This study included all TNBC cases seen and handled between January 2016 and December 2020. TNBC patients' information was gathered and distributed depending on their grade, histological type of breast cancer, stage, and stage group, as determined by biopsy, blood testing, computed tomography (CT), cytology, histology, and/or magnetic resonance imaging (MRI). Patients with mental impairments who made adequate follow-up and reporting impossible, as well as those with insufficient information, were eliminated from the study. The total number of TNBC cases was 127 between January 2016 and December 2020. Formalin-fixed paraffin-embedded (FFPE) tissue blocks of only 53 cases out of 127 TNBC cases were included in this study because many Iraqi breast cancer patients used to travel outside Iraq for management and used to take their tissue blocks with them for further molecular studies. We used a formula created specifically for this study to extract sociodemographic, clinical, and histopathological characteristics. Tumor size, tumor grade, disease stage, the potential of recurrence, and the existence of distant metastases were all clinical factors. It is worth noting that some of the information in the database came directly from patients and, in a few cases, first-degree relatives who were working as caretakers. Excel sheets were produced with a research number for each case, and all the clinicopathological parameters were gathered in the formulated Excel sheet.

### Immunohistochemistry and interpretation

**Figure 2:**
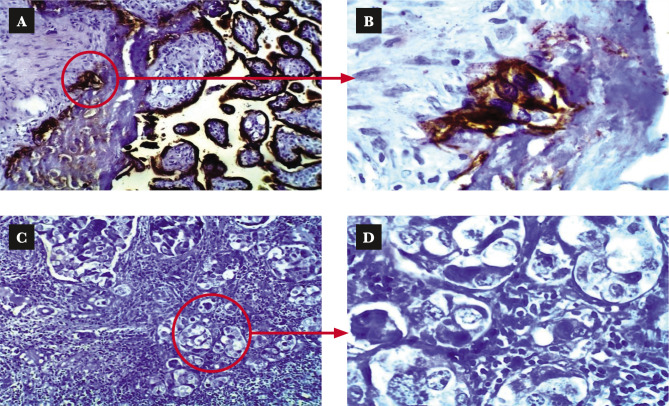
Positive and negative control slides. A – Positive control slides from BIOSB show colorectal adenocarcinoma with strong positive EGFR membranous brown staining with cytoplasmic performance. Objective 10X. B – The same as (A) at objective 40X. C – negative control slide, the section of breast carcinoma with omitting of the primary antibody. D – The same as (C) at objective 40X.

**Figure 3:**
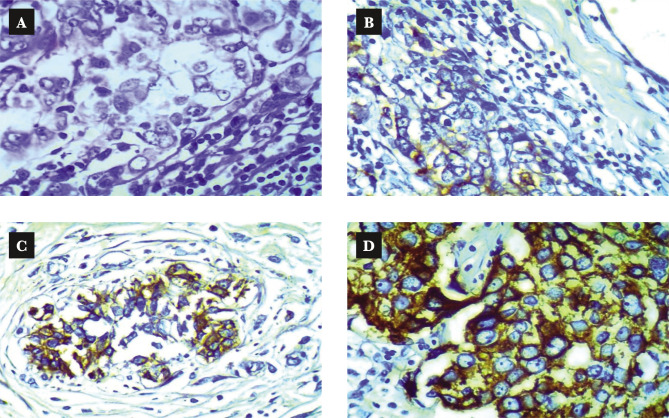
Scoring of EGFR in TNBC. A –Score 0; B – Score +1; C – Score +2; D – Score +3.

**Figure 4:**
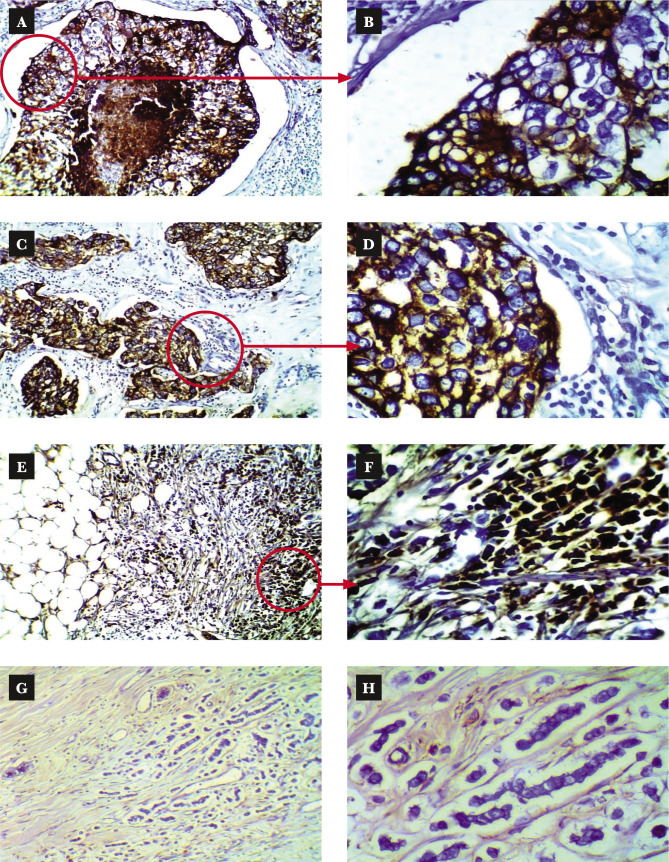
EGFR immunohistochemical expression in TNBC. A – Section of TNBC with DCIS with central necrosis shows strong positive staining for EGFR. Objective 10X. B – The same section (A) at objective 40X shows the strong membranous brown staining for EGFR with cytoplasmic performance. C – Section of TNBC with invasive ductal carcinoma shows strong positive staining for EGFR. Objective 10X. D – High power field of (C) at objective 40X. E – Section of TNBC with invasive lobular carcinoma shows strong positive staining for EGFR. Objective 10X. F – High power field of (E) at objective 40X. G – Section of TNBC with invasive lobular carcinoma shows negative staining for EGFR. Objective 10X. H – High power field of (G) at objective 40X.

The immunohistochemistry staining was performed using the Dako EnVision detection immunohistochemistry kit (Envision FLEX, Dako, K8000, Denmark) and as per the manufacturer's instruction. Anti-epidermal growth factor receptor primary antibody (monoclonal Mouse Anti-Human Epidermal Growth Factor Receptor (EGFR), Dako, M3563, Denmark) was used to detect EGRF expression in this study. The primary antibody was diluted 50 fold with antibody diluent (EnVision FLEX Antibody Diluent, Dako, K8006, Denmark). EGFR positive control slides (EGFR control slide, BioSB, BSB 5476, Netherland) were used to confirm the procedure, where one positive control slide proceeded for each time the IHC procedure (Figure 2 A, B). We used the same tissue sections for the negative control omitting the primary antibody (Figure 2 C, D). Immunostaining interpretation was made using a bright-field Leica microscope. The stained slides were interpreted by a pathologist manually, as shown in Table 1 [15] and Figure 3 A–D. EGFR overexpression was seen in different histopathological types of TNBC (Figure 4 A–H).

**Table 1:** Immunohistochemical scoring system of EGFR.

Score	Interpretation
0	No staining or weak membranous staining
+1	Weak membranous staining in ≥10% of the tumor cells
+2	Moderate, membranous staining in ≥10% of the tumor cells
+3	Strong membranous staining in ≥10% of the tumor cells

The primary objective of this study was to investigate the clinical significance of EGFR expression in association with other clinicopathological parameters in TNBC.

### Statistical analysis and machine learning techniques

For statistical analysis, we used Python3 scipy software, version 1.4.1 for correlations (Pearson & Spearman). For PCA (principal component analysis), we used Python3 sklearn, version 0.20.3. Plots and Roc (Receiver Operator Characteristic) curves were produced using Matplotlib and seaborn software version 3.6.9. P-values of <0.05 were considered statistically significant. Principal component analysis (PCA) is a technique used to reduce the dimensionality of the datasets, clarify the data, and decrease the risk of loss of data information [16]. The receiver operator characteristic (ROC) curve represents the relationship between the actual positive rate (sensitivity) and the false positive rate (100-specificity) at various cut-off positions for a parameter. Each point on the ROC curve corresponds to a pair of sensitivity/specificity values associated with a specific decision threshold. The area beneath the curve (AUC) provides a snapshot of a model's performance at various threshold settings [17].

## Results

### Demographic distribution

Table 2 shows that the total number of TNBC patients we collected over the last five-year period was 127. The median age was 50, ranging from 25 to 88 years old. Most TNBC patients were younger than 60; 96 (75.6%) and 31 (24.4%) were 60 years old and above. Table 2 shows the percentage of tumor histopathological types; most patients, 102 (80.4%), had invasive ductal carcinoma. Regarding the clinical stage, most patients had stage II tumors – 60 (47.2%). Distant metastasis was present in 24 (19%) of reported cases. The recurrence of the tumor was reported in 8(6.3%) patients only. Of the total of 127 patients with TNBC, we collected the FFPE tissue blocks of 53 patients to evaluate the EGFR expression by immunohistochemistry. The demographics of the 53 cases studied for EGFR expression show the same median age and range as the original TNBC data. Again, 42 (79.25%) patients were younger than 60 years old, and 11 (20.75%) were 60 years old or above. 33 patients had (62.4%) invasive ductal carcinoma for the tumor histopathological types. Regarding the grading of the tumor, most of the patients had grade III 27 (51%). Stage II was the most typical clinical stage identified among the involved cases 29 (54.75%). Distant metastasis occurred in 11(20.75%) patients. Tumor recurrence was reported in just 2 (4%) patients. EGFR expression was positive in 15 (28%) patients and negative in 38 (72%). 42 patients are still alive (79%), 8 (15%) people deceased, and 3 (6%) patients have unknown status (Table 3).

**Table 2:** Demographic characteristics of the study population (127 TNBC patients).

Category	No. (%)
Total no.	127
Age	
**<60**	96 (75.6)
**>60**	31 (24.4)
**Median age**	50 years
**Age range**	25–88 year
Histopathologic types	
**Invasive ductal carcinoma**	102 (80.4)
**Invasive lobular carcinoma**	5 (3.9)
**Medullary carcinoma**	6 (4.7)
**NOS**	14 (11)
Tumor grade	
**II**	60 (47)
**III**	56 (44)
**Unknown**	11 (9)
Clinical stage	
**I**	2 (1.5)
**II**	60 (47.2)
**III**	28 (22)
**IV**	24 (19)
**Unknown**	13 (10.3)
Distant metastasis	
**Present**	24 (19)
**Absent**	103 (81)
Recurrence	
**Present**	8 (6.3)
**Absent**	117 (92.1)
**Unknown**	2 (1.6)

**Table 3:** Demographics of the 53 TNBC cases included in EGFR immunohistochemical expression analysis.

Category	No. (%)
Total no.	53
Age	
**<60**	42 (79.25)
**>60**	11 (20.75)
**Median age**	50 years
**Age range**	28–88 year
Histopathological subtypes	
**Invasive ductal carcinoma**	33 (62.4)
**Invasive lobular carcinoma**	4 (7.5)
**Medullary carcinoma**	2 (3.7)
**NOS**	14 (26.4)
Tumor grade	
**II**	24 (45)
**III**	27 (51)
**Unknown**	2 (4)
Clinical stage	
**I**	0
**II**	29 (54.75)
**III**	9 (17)
**IV**	11 (20.75)
**Unknown**	4 (7.5)
EGFR expression	
**Positive**	15 (28)
**Negative**	38 (72)
Survival	
**Alive**	42 (79)
**Deceased**	8 (15)
**Unknown**	3 (6)
Distant metastasis	
**Present**	11 (20.75)
**Absent**	42 (79.25)
Recurrence	
**Present**	2 (4)
**Absent**	50 (94)
**Unknown**	1 (2)

### Univariate correlation analysis

Univariate correlation analysis was used to measure the association between the clinicopathological variables and test the association between EGFR expression and other clinicopathological parameters. Pearson's correlation analysis was applied for the TNBC data of 127 cases and the 53 cases with EGFR expression analysis (Table 4). For the 127 TNBC cases, we found a highly significant negative correlation between distant metastasis and recurrence. The Pearson coefficient value was -0.45, and the p-value was <0.000001. There was a highly significant negative correlation between the clinical stage of the tumor and the recurrence. The Pearson coefficient value was -0.42, and the p-value<0.000001. On the other hand, there was a highly significant positive correlation between the clinical stage and the distant metastasis, with a Pearson coefficient value of 0.84 and p-value<0.000001. There was no significant correlation between the other clinicopathological parameters. Table 5 shows the Pearson correlation and the P-value for the 53 TNBC cases analyzed by immunohistochemistry for evaluating EGFR expression. Interestingly there was a significant positive correlation between EGFR expression and death. The Pearson coefficient value was 0.55 and the p-value 0.00004, and the correlation was significantly positive between death and the clinical stage, Pearson coefficient value of 0.41 and p-value 0.004. There was a significant positive correlation between the clinical stage and distant metastasis, the Pearson coefficient value was 0.88, and the p-value was highly influential <0.00001. Moreover, there was a significant positive correlation between the death of the patients and distant metastasis; the Pearson coefficient value was 0.43, and the p-value 0.002. There was no significant correlation between the other clinicopathological parameters.

**Table 4:** Pearson correlation analysis between the clinicopathological variables. The correlation coefficient and its p-value for the 127 TNBC cases.

Variable 1	Variable 2	Correlation Coefficient	p-value
Age	Grade	0.05	0.6
Age	Recurrence	-0.25	0.005
Age	Distant metastasis	0.08	0.4
Age	Clinical stage	0.09	0.3
Grade	Recurrence	-0.18	0.05
Grade	Distant metastasis	0.15	0.1
Grade	Clinical stage	0.18	0.06
Recurrence	Distant metastasis	-0.45	<0.000001*
Recurrence	Clinical stage	-0.42	<0.000001*
Distant metastasis	Clinical stage	0.84	<0.000001*

**Table 5:** Pearson correlation analysis for the 53 TNBC patients included in EGFR immunohistochemical expression analysis and p-values.

Variable 1	Variable 2	Correlation Coefficient	p-value
EGFR	Age	-0.01	0.96
EGFR	Grade	0.05	0.72
EGFR	Recurrence	-0.20	0.17
EGFR	Distant Metastasis	0.30	0.03
EGFR	Clinical Stage	0.29	0.04
EGFR	Death	0.55	0.00004
Age	Grade	0.11	0.54
Age	Recurrence	0.01	0.94
Age	Distant Metastasis	-0.03	0.8
Age	Clinical Stage	-0.04	0.8
Age	Death	0.14	0.33
Grade	Recurrence	-0.23	0.12
Grade	Distant Metastasis	0.23	0.1
Grade	Clinical Stage	0.20	0.16
Grade	Death	0.03	0.82
Recurrence	Distant Metastasis	-0.10	0.47
Recurrence	Clinical Stage	-0.20	0.17
Recurrence	Death	-0.03	0.84
Distant Metastasis	Clinical Stage	0.88	<0.00001
Distant Metastasis	Death	0.43	0.002
Clinical Stage	Death	0.41	0.004

### Multivariate prediction analysis model using machine learning

We investigated the risk of death, distant metastasis, and recurrence for TNBC patients using the available clinicopathological parameters (age, grade, clinical stage, recurrence, and distant metastasis) as inputs for the PCA model, including EGFR expression data. The PCA model was used to predict the risk of death among TNBC patients, whereby EGFR expression data and other clinicopathological parameters were used as inputs for this model.

**Figure 5:**
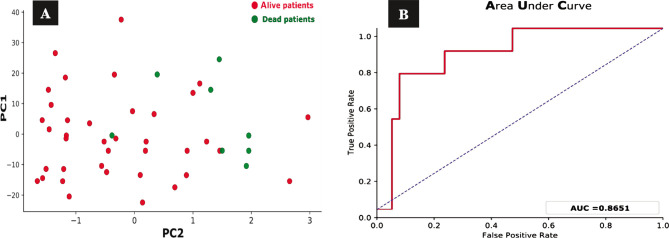
Principal component analysis (PCA) cluster plot and ROC curve for the death prediction. A – PCA shows the cluster of green dots on the right side (the area where the people are at high risk of death), while the red dots on the left side (the area where the people are at low risk of death). Two dead patients (green dots) on the left side of the plot represent false-positive data, and the red dots on the right side are the false-negative results. B – ROC model for the PCA model to predict death for TNBC patients. The area under the curve is about 0.87 (87%).

**Figure 6:**
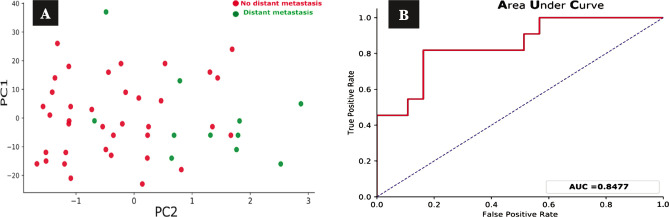
Principal component analysis (PCA) cluster plot and ROC curve for the prediction of distant metastasis. A – The cluster plot shows the green dots that represent the patients with distant metastasis more on the right side of the figure, while most of the red dots, which represent the patients with no distant metastasis, are clustered more on the left. B – ROC model for the PCA model to predict distant metastasis for TNBC patients. The area under the curve is about 0.85 (85%).

**Figure 7:**
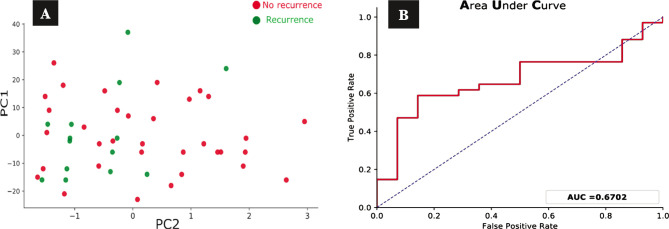
Principal component analysis (PCA) cluster plot and ROC curve for the prediction of recurrence. A – No clear distinction between the green dots representing the patients who develop recurrence and the red dots representing the patients with no recurrence distributed all over the figure and in no clear clusters. B – ROC model for the PCA model to predict recurrence for TNBC patients. The area under the curve (AUC) is 0.67 (67%).

The cluster plot clearly distinguishes the dead patients represented by the green dots (Figure 5A) and the alive patients defined by the red dots (Figure 5B) and shows the validity test performed to validate the PCA model to investigate the false positive and the false-negative results in numbers. To achieve that validity test for our model, we used the receiver operating characteristic (ROC) model. The area under the curve was 0.87, which means the classifier had excellent validity to distinguish between the true positive and the false-positive rates and, ultimately, a better classifier. We used PCA to predict the risk of distant metastasis among TNBC patients. EGFR expression data and other clinicopathological parameters were used as inputs for this model, excluding the death parameter. Figure 6A shows that most patients with distant metastasis, represented by the green dots, are on the right side, while the red dots represent most patients with no distant metastasis. This means that the risk of distant metastasis increases in the presence of other collective factors like high tumor grade, advanced clinical stage, and positive EGFR expression. The area under the curve was about 0.85, which is considered close to the perfect curve, and this is also shown in the ROC system (Figure 6B). This means our model has high validity in predicting distant metastasis. The predicting risk of recurrence among TNBC patients involved EGFR expression data and other clinicopathological parameters used as inputs for this model, excluding death and distant metastasis. For assessing the risk of recurrence for TNBC patients using the PCA model, Figure 7A reveals no clear distinction between those at high risk of recurrence, represented by the green dots, and those at low risk of recurrence. Figure 7B shows that the AUC was 0.67, close to the random classifier, unlike the previous death and distant metastasis prediction results.

## Discussion

The incidence of breast cancer in Iraq and worldwide is still at the top of malignancies among women, and its morbidity is still high and out of clinical control [18]. Triple-negative breast cancer is a highly heterogeneous subtype of breast cancer at molecular and genetic levels with a worse prognosis and poor survival than other breast carcinoma subtypes. Understanding the relationship between molecular targets and different clinicopathological parameters of TNBC patients is focused on many recent studies to explore more about prognostic and predictive biomarkers. EGFR is one of the promising targets in TNBC and the focus of many recent projects. EGFR is more frequently overexpressed in TNBC than in other breast cancer subtypes, and EGFR expression has been acknowledged as a weak prognostic marker for TNBC [19]. EGFR expression, gene amplification, and mutation status have been extensively researched in TNBC; however, we were unable to locate any local study demonstrating the EGFR expression profile in TNBC. Additionally, the FDA previously rejected EGFR as a targeted treatment for TNBC patients. The hypothesis of this project was based on investigating the profile of EGFR immunohistochemical expression in a series of Iraqi patients and the clinical significance of this marker in TNBC by focusing on studying the association between EGFR expression and the clinicopathological parameters of TNBC, including the death of patients.

To achieve the above objectives of our study, we applied correlation tests and modern analysis tools using machine learning. We used EGFR expression data and other clinicopathological parameters as inputs of the machine learning model (PCA and ROC curve). We found an interesting significant positive correlation between EGFR overexpression and death events of TNBC patients and the possibility of using EGFR overexpression with other clinicopathological factors as a predictive tool to predict the prognosis and outcome of TNBC patients. Positive EGFR expression in patients with TNBC in our study was 28%, whereas Weihua Jiang *et al*. found that the EGFR expression in patients with TNBC was 45% [20]. Choi *et al*. [13] and Rakha *et al*. [21] reported a frequency of 13% and 37% of EGFR overexpression, respectively. Tan *et al*. reported a rate of 52% of EGFR overexpression in TNBC [22]. Martin *et al*. showed a frequency of 76% of EGFR overexpression in TNBC [23]. All these differences in the rate of EGFR expression may be explained by the difference in the antibody used, the method of evaluation, the cut-off values for EGFR, ethnicity, and also by the total number of cases in each study. In our study, TNBC was more common among young females, and the median age was 50 years. Shawn *et al*. found that the mean age at the time of diagnosis for TNBC patients was 55.5±13.1 years [24]. Boyle P, in his research, found that women with TNBC were significantly more likely to be under the age of 40 [25].

Our possible explanation for this observation could be that breastfeeding is a well-known protective factor against breast cancer in general and also against TNBC [26], and in recent years, many mothers preferred bottle feeding, which was less used among older females in their youth. Furthermore, our data showed that most female patients affected with TNBC had an invasive ductal carcinoma (approximately 80%). In their research, Liao *et al*. found that invasive ductal carcinoma affected approximately 91% [27]. Sanges *et al*. achieved a prevalence of invasive ductal carcinoma in TNBC of about 78% [28]. This could be explained by invasive ductal carcinoma being the most common and aggressive breast cancer. Accordingly, TNBC, one of the most aggressive breast cancers, is more common among these patients [29]. We also noticed that most patients with TNBC had a stage II tumor (47%), and most had no distant metastasis (81%). The same results were noticed by Silvana *et al*. [28] as they found that of their total number of patients with TNBC, 46.5% had a stage II and about 95.8% had no distant metastasis. From our point of view, the fact that most patients with TNBC were stage II at diagnosis may be related to the increased awareness of frequent self-examination of the breast due to the global influence of programs designed for early detection of breast cancer. In contrast, Qiu *et al*. found that distant metastasis was present in about 20% of the total number of patients with TNBC [30]; the reason behind this difference may be related to the difference in the sample size of patients in each study.

Regarding the tumor grade, most patients with TNBC in our study had a histologic grade II (47%) and grade III in 44%. Park *et al*. showed nearly the same results in that 51% of their patients had grade III, and 47% had grade II [31]. Li *et al*. found that grade II constitutes about 42% of their patients, whereas grade III constitutes about 34% [32]. These differences may be related to the difference in the sample size and the time between the beginning of the cancerous changes and the diagnosis. In our study, only 6% of patients with TNBC developed tumor recurrence, and Li *et al*. also found a low recurrence rate of about 10%. In contrast, Pogoda *et al*. found that one-third of their patients developed tumor recurrence over six years of observation. These highly different findings could be explained by the difference in the sample size among these studies, the genetic differences between the studied populations [33], the type and duration of treatment given to the patients [34], the elapsed time between the diagnosis and the time of the study (period of observation).

### Univariate correlation analysis

In this part of our results, we did a univariate correlation analysis to investigate the correlation between the clinicopathological parameters and EGFR expression. We found a significant positive correlation between death and the clinical stage. The same finding was achieved by Suresh *et al*. as they discovered a significant difference in RFS (recurrence-free survival) for the pathological stage of disease (P=0.05). The three-year RFS for stage II and III patients was 70% and 50%, respectively [35]. Additionally, a strong positive connection was discovered between death and distant metastases. In comparison, Chen *et al*. discovered that age at diagnosis, race, T stage, molecular subtypes, surgery, radiation therapy, and distant organ metastasis were all linked with breast cancer-specific survival (BCSS) (P=0.05). Except for bone metastases (P=0.299), all of the variables listed above were associated with overall survival (OS) (P=0.05) [36]. Interestingly, we found a significant positive correlation between the death event and EGFR expression.

Jiang *et al*. [20] found that compared with EGFR-negative cases, those with positive EGFR expression indicated a log-rank value of 11.864 and P<0.01 [20]. This implies that patients with positive EGFR expression had a poorer prognosis than those with negative EGFR expression. Our and their findings indicate that patients with positive EGFR expression have a shorter life expectancy than those with negative EGFR expression [20]. This may be explained by the fact that survival is affected by many factors, including the clinical stage, recurrence, and distant metastasis. These factors are closely associated with the positive expression of EGFR, where EGFR expression could play a role in the mechanistic behind the tumor progression and metastasis, increasing the chance of death. Moreover, we found a significant positive correlation between the clinical stage and the distant metastasis in the 127 TNBC cases and 53 patients on whom we evaluated the EGFR expression. Yi *et al*. achieved somewhat the same results as they found that age >50, clinical-stage III-IV, higher stage, and tumor size >5 cm were independent risk factors for distant metastasis of primary TNBC [37]. We found that for the total of 127 TNBC cases, there was a negative correlation between the clinical stage and the distant metastasis with the recurrence risk; the p-value was 0.0000001 and 0.000004, respectively, which was a weak correlation. This can be explained by the fact that most patients with high clinical stage and/or those who developed distant metastasis may have died before they developed a recurrence.

### Multivariate prediction analysis using machine learning

Machine learning (ML) is a modern tool to analyze and interpret data. Several published articles utilize ML approaches to predict several types of cancers. For breast cancer susceptibility, Ayer *et al*. demonstrated that machine learning could effectively assess the risk of breast cancer using a dataset combining demographic data and prospectively recorded mammographic findings [38]. Listgarten *et al*. stated that in order to investigate the effect of genetic polymorphisms on breast cancer risk, they used machine learning techniques to identify a subset of genetic variations as significant discriminators between breast cancer and controls [39].

For breast cancer recurrence, Kim *et al*. used machine learning algorithms to compare the breast cancer recurrence prediction based on ML and the traditional Nottingham prognostic index (NPI). ML outperformed all other algorithms. These findings indicate that machine learning may be a powerful tool for predicting breast cancer recurrence [40]. Ahmad *et al*. concluded that various data mining approaches might be utilized to forecast breast cancer recurrence. They studied breast cancer data and compared the outcomes using three categorization algorithms for predicting cancer recurrence. The findings suggested that machine learning is a compelling method for predicting breast cancer recurrence [41]. Park *et al*. discovered that the semi-supervised learning model best predicts breast cancer patient deaths. It demonstrated a high degree of total accuracy and stability [42]. Xu *et al*. found a 50-gene signature and improved prediction performance by 34%, 48%, and 3%, respectively, compared to the commonly used 70-gene signature [43].

In our study and with the aid of a multivariate prediction analysis model using machine learning, we were able to predict the risk of death, the risk of distant metastasis, and the risk of tumor recurrence by using EGFR expression data and their clinicopathological parameters like age, tumor grade, clinical stage, distant metastasis, recurrence, and death event as inputs for our model. Interestingly, the PCA model shows a clustering of our patients into two groups distinct from each other for death prediction. The cluster of the patients with advanced-stage, high tumor grade, distant metastasis, and positive EGFR expression represent patients at high risk of death (dead patients). The other cluster with lower stage and more differentiated tumor grade, no distant metastasis, and negative EGFR expression represent patients at low risk of death (alive patients). This model's validity and power were tested using the receiver operator characteristic (ROC) curve. The area under the curve (AUC) metric indicates a model's ability to discriminate across classes and summarize the ROC curve. The greater the AUC, the more accurate the model, distinguishing between positive and negative classifications. For the death prediction, AUC was about 87%. When the AUC increases and gets close to 1, the classifier has excellent validity to distinguish between the true positive and the false-positive rates and ultimately becomes a better and more robust classifier. This validity test supports the reality and the power of the prediction model we used in our study.

Using the above approach to deal with EGFR expression data for TNBC cases opens the door for the possible future implication of such a model in clinical practice. We further explain the false-positive results in our model, which were very few and not significant. From our point of view, those who were at low risk of death and present in the area of dead patients in the cluster figure (Figure 5A) may have died because of other conditions that made them at risk of death, like the low level of medical care, comorbid conditions, inability to afford the required investigations and drugs and the need for special treatment options that are not available in Iraq. Yucan *et al*., in their study, discovered that machine learning modeling techniques could generate more accurate prognostic models for 5-year mortality outcomes in patients with TNBC [44]. Although it used different clinicopathological parameters and different ML tools, this study found the utility of ML in the prediction of death in TNBC. Regarding the prediction model of distant metastasis, our study revealed that the patients were distributed in distinct clusters. The cluster of patients with advanced-stage, high tumor grade, and positive EGFR expression represent patients at high risk of distant metastasis.

The other cluster with lower stage and more differentiated tumor grade and negative EGFR expression represent patients at low risk of distant metastasis. The ROC curve for the distant metastasis prediction model supports the finding of our PCA model as the AUC is about 85%. For the prediction of the recurrence risk, our study did not find an apparent association between the clinicopathological parameters and EGFR expression in predicting the risk of recurrence, as shown previously for death and distant metastasis.

The possible reason for such findings may be the limited time for follow-up of the patients by the tertiary center from where most of our data come, the early death also decreases the chance of recurrence of TNBC patients in the future, and finally, the small sample size. Using this analysis model for our data, we were able to frame new information about the clinical significance of EGFR expression in TNBC by predicting the risk of death and distant metastasis. Thereby, this model may help decide which group of patients have a low risk of death and distant metastasis, and ultimately the patient may benefit from the limited health resources in some countries like Iraq and direct these resources to people who will benefit the most.

Given our findings, it may be of high benefit for the physicians and oncologists to take into consideration that the risk of death and/or the risk of distant metastasis may be predicted by evaluating the EGFR status of the patients in correlation with other clinicopathological parameters like the tumor stage, tumor grade, lymph node status, and others. Studying all these parameters using machine learning models can provide a clear view of their patient's prognosis and hence improve the outcome of TNBC patients.

## Conclusions

This study provides new information about the clinical significance of EGFR expression in TNBC in the Middle Euphrates area of Iraq, focusing on its relationship with the clinicopathological parameters. EGFR is expressed in about 28% of patients with TNBC, and its expression is associated with poor prognosis and an increased risk of death. The advanced clinical stage of the tumor is associated with a higher risk of distant metastasis and death. The death risk is also increased with the presence of distant metastasis. Machine learning programs are an excellent tool to approach EGFR expression data in patients with TNBC to build a model. It is possible to predict the risk of death and distant metastasis using EGFR expression data with other clinicopathological characteristics. Such models could pave the way for new strategies to improve survival in TNBC patients and help direct the limited health resources in developing countries like Iraq and build up a cost-effective therapeutic approach for cancer patients in general and those with aggressive breast cancer malignancies especially those with TNBC. In the upcoming years, evaluation of EGFR expression can be utilized as an independent prognostic tool and a target for studying the treatment options for TNBC.

There are a few limitations, especially concerning the sample size and difficulties in getting tissue blocks of breast cancer patients because most of our patients traveled outside Iraq for molecular diagnosis and therapy, which could affect the generalizability. The application of Fluorescent InSitu Hybridization in a future study to correlate the positive EGFR IHC with FISH to understand the sensitivity of the IHCs and the technique used could pave the way for future application of EGFR detection by IHC in TNBC as a routine clinical test. Lack of survival, molecular, and genetic data would help understand the behavior and relationship between EGFR expression in TNBC in these areas and could provide a powerful resource for designing predictive artificial intelligence and machine learning models for predicting different molecular types of breast cancer and their outcomes.
